# Preadministration of Fermented Sorghum Diet Provides Protection against Hyperglycemia-Induced Oxidative Stress and Suppressed Glucose Utilization in Alloxan-Induced Diabetic Rats

**DOI:** 10.3389/fnut.2018.00016

**Published:** 2018-03-12

**Authors:** Tolulope Dorcas Olawole, Margaret Imuetiyan Okundigie, Solomon Oladapo Rotimi, Ogi Okwumabua, Israel Sunmola Afolabi

**Affiliations:** ^1^Department of Biochemistry, College of Science and Technology, Covenant University, Ota, Nigeria; ^2^Department of Pathology and Population Medicine, Midwestern University, Glendale, AZ, United States

**Keywords:** diabetes mellitus, sorghum, antioxidants, gene expression, nutrition, glycolytic enzymes, oxidative stress

## Abstract

*Sorghum bicolor* grains are rich in phytochemicals known to considerably impact human health. Several health-promoting products such as flour, staple food, and beverages have been produced from sorghum grains. This study investigated the protective and modulatory effects of a sorghum diet on the genes of some antioxidant and glycolytic enzymes in alloxan-induced diabetic rats. The rats were randomly distributed into six groups: the control group received normal diet, while the other groups were pretreated with 12.5, 25, 50, 75, and 100% of the sorghum diets daily for 8 weeks before the administration of a dose of alloxan (100 mg/kg BW), after which blood was collected and the liver was excised. The effects of the diets on blood glucose levels, liver dysfunction indices, and markers of oxidative stress were assessed spectrophotometrically, while the gene expressions of key glycolytic enzymes and enzymatic antioxidants were assayed using reverse transcriptase polymerase chain reaction. It was observed that the pretreatment of the experimental animals with the diets normalized the blood glucose before and after the administration of alloxan. The sorghum-treated groups also showed statistically significant (*p* < 0.05) decrease in liver dysfunction indices and markers of oxidative damage compared with the control. In addition, statistically the diets significantly decreased (*p* < 0.05) the relative expression of superoxide dismutase, glutathione peroxidase, glucokinase, phosphofructokinase, and hexokinase genes in the experimental animals compared with the control. Overall, this study showed that the preadministration of fermented sorghum diet significantly protected against hyperglycemia and suppressed glucose utilization *via* glycolysis in the liver of alloxan-induced diabetic rats. Thus, the consumption of sorghum diet may protect against hyperglycemia and oxidative damage and may therefore serve as functional food for management of diabetic mellitus.

## Introduction

Diabetes mellitus (DM) is a metabolic disorder characterized by elevated levels of glucose in the blood resulting from defects in the action or secretion of insulin (1). According to the International Diabetes Federation (2015) (2), 1 in every 11 adults has diabetes, and over one million children have type 1 diabetes. The increasingly high global prevalence (6.4%) of diabetes in 2010 was projected to reach 69 and 20% among adult in the developing and developed world, respectively, by the year 2030 (3). Type 1 DM also known as juvenile diabetes is characterized by a total deficiency of insulin due to the autoimmune destruction of the pancreatic beta cells (1). This is characterized with disturbances of carbohydrate, lipid, and protein metabolism, resulting in the alterations in enzymatic activities of metabolic pathways such as gluconeogenic, glycolytic, lipolytic, and pentose phosphate pathways (4). Insulin, the major regulator of glucose uptake into the cells, depends largely on glucose transporters. The absorption of glucose activates hepatic glucokinase (GK), which in turn activates the rate limiting enzyme of glycolysis—phosphofructokinase-1 (PFK-1)—allowing glucose to be rapidly oxidized to pyruvate. In the absence of this, a prolonged elevated blood glucose level promotes lipid peroxidation and subsequently enhances the production of reactive oxygen species (ROS), thus damaging macromolecular components and compromising the antioxidant defense system (5). The prevalence of DM has led to a growing interest in natural remedies and dietary interventions, which are safer alternatives in the management of the disease due to the side effects associated with several antidiabetic medications (6).

*Sorghum bicolor* is a grain that serves as food for both animals and humans. Sorghum is rich in phytochemicals that have been reported to have glucose-lowering (7) and cholesterol-lowering properties (8). Scientific evidence has also shown that sorghum extracts has hypoglycemic activity in diabetic rats, thus helping to control the negative effects of DM (9, 10). The process of fermentation of foods has numerous nutritional and health benefits such as improvement in flavor, appearance, texture, nutritional value and palatability through increased bioavailability of nutrients, increased carbohydrate digestibility, and shelf-life extension (11, 12). A fermented cereal also known as “*ogi*” or “*oka-baba*” is a staple and indigenous food produced from sorghum, millet (*Pennisetum americanum*), maize (*Zea mays*), or guinea corn (*Sorghum spacers*). It is consumed by both the young and old and particularly used as a weaning food for children (12, 13). The phytochemical status of unfermented sorghum grains (13, 14) and the fermented sorghum grains (15) has earlier been reported. Caffeic acid, a major phytochemical constituent of fermented sorghum, has been implicated in the treatment of diabetes (15). The traditional application of fermented sorghum grains has been attributed to its antioxidant and antibacterial potential in the treatment of diarrhea (16). Although analysis of the extract of “*ogi*” revealed the presence of several important bioactive compounds such as quercetin, caffeic acid, hesperidin, and rosmarinic acid among a host of others, scientific evidence on the hypoglycemic effect of this fermented cereal is lacking. Understanding the mechanisms of action of the hypoglycemic properties of sorghum diet will enhance the knowledge of providing further solution to diabetes. The high cost of antidiabetic drugs equally demands for alternative approaches that include nutritional approach to prevent the disease especially in developing countries. Therefore, in this study, we investigated the protective effects of a fermented sorghum diet (FSD) on the modulation of hepatic glucose metabolism in alloxan-induced type 1 diabetic rats by assessing its influence on blood glucose levels, biomarkers of oxidative stress, liver dysfunction indices, and hepatic mRNA expression of both glycolytic enzymes and enzymatic antioxidants.

## Materials and Methods

### Chemicals

Folin–Ciocalteu’s reagent, 2,2-diphenyl-1-picrylhydrazyl, 5,5-dithiobis nitrobenzoic acid, 1-chloro-2,4-dinitrobenzene, glutathione, hydrogen peroxide (H_2_O_2_), pyrogallol, trichloroacetic acid, potassium dihydrogen phosphate (KH_2_PO_4_), dipotassium hydrogen phosphate (K_2_HPO_4_), and 2-thiobarbituric acid were products of Sigma-Aldrich (St. Louis, MO, USA). The polymerase chain reaction (PCR) primers were purchased from Life Technologies (MD, USA). All other chemicals were of analytical grade.

### Animals and Diet

Healthy female Wistar albino rats weighing 150–200 g were purchased from the Federal University of Agriculture, Abeokuta, Ogun State, Nigeria. The animals were accommodated in well-ventilated cages at room temperature for 4 weeks to allow for their adaptation. The rats were fed with standard rodent diet and water *ad libitum*. This study was carried out in accordance with the recommendations of ethical committee of Covenant University, Ota, Nigeria, with the reference number CU/BIOSCRECU/BIO/2015/010. The protocol was approved by the ethical committee of Covenant University.

*Sorghum bicolor* grains were purchased directly in Oyo State, Nigeria (7°19′60 ″ N and 4°4′0″ E), and the cleaning and fermentation was carried out (15). The resulting extract was filtered and oven dried at 40°C for 6 h to obtain a dried fermented sorghum flour (FSF) (17). Semipurified FSDs were prepared (18, 19) to contain 12.5, 25, 50, 75, and 100% FSF (Table 1). Each flour mix was immediately compressed into a cylindrically shaped cake before air drying.

**Table 1:** The composition of experimental diet.

Component	Levels (g/100 g in diet)					
	
	Control	12.5% FSD	25% FSD	50% FSD	75% FSD	100% FSD
Cornstarch	50	42.5	35	20	5	–
Fermented sorghum	–	7.5	15	30	45	50
Sucrose	10	10	10	10	10	10
Cellulose	5	5	5	5	5	5
Groundnut oil	5	5	5	5	5	5
Fish meal	26	26	26	26	26	26
Vitamin/mineral mix^a^	4	4	4	4	4	4

*^a^Vitamin/mineral mix contains the following (in g = 100 g): calcium phosphate, 49.50; sodium powder, 11.80; potassium sulfate, 5.20; sodium chloride, 7.40; magnesium oxide, 2.40; potassium citrate, 22.40; ferric citrate, 0.60; manganese carbonate, 0.35; cupric carbonate, 0.03; zinc carbonate, 0.16; chromium potassium sulfate, 0.055; potassium iodate, 0.001; sodium selenate, 0.001; choline chloride, 0.50; thiamine HCl, 0.06; riboflavin, 0.06; niacin, 0.30; calcium pantothenate, 0.16; biotin, 0.01; vitamin B12, 0.10; vitamin D3, 0.025; vitamin E acetate, 1.00; pyridoxine, 0.07; folic acid, 0.02; vitamin A acetate, 0.08*.

*FSD, fermented sorghum diet*.

### Experimental Design

The rats were divided into six groups with each group consisting of six rats. Group 1 served as the control and received no sorghum diet, while groups 2, 3, 4, 5, and 6 received 12.5, 25, 50, 75, and 100% the sorghum diet, respectively, for 8 weeks before the induction of diabetes.

### Induction of Experimental Diabetes

At the end of the dietary regimen, a freshly prepared solution of alloxan monohydrate (100 mg/kg BW) in sterile normal saline (0.9% NaCl) was injected intraperitoneally to the overnight fasted rats in all the groups (20). The rats were given 10% glucose solution after induction of DM to avoid drug-induced hypoglycemia.

### Biochemical Analysis

Blood samples were collected by nipping the tails of the rat before and after induction of DM. Blood glucose was immediately measured by glucose strips using the Accu-check^®^ advantage glucose meter (Roche Diagnostics Co., USA). The rats were sacrificed after an overnight fast, and blood was collected by cardiac puncture. Blood and liver samples was processed and centrifuged at 3,000 *g* for 10 min, and the homogenate was stored at −20°C for biochemical analysis. A portion of the liver was also preserved in RNAlater for molecular analysis. The activities of aspartate transaminase (AST), alanine transaminase (ALT), alkaline phosphate (ALP), and total protein in the liver were measured using the respective commercial kits (Randox Inc., UK).

### Determination of Biomarkers of Oxidative Stress

The concentrations of thiobarbituric acid reactive substances, peroxidase (Px), and superoxide dismutase (SOD) were assessed using the method described by Ohkawa et al. (21), Chance and Maehly (22), and Magwere et al. (23), respectively.

### Expression of Hepatic Glycolytic and Antioxidant Genes

The levels of expression of certain hepatic glycolytic genes were assessed using semiquantitative reverse transcriptase polymerase chain reaction (RT-PCR) as previously described by Atangwho et al. (24). Briefly, RNA was isolated from the liver samples using the spin column kit obtained from Aidlab’s EASYspin Plus^®^ (Aidlab Biotechnologies Co., Ltd., Beijing, China) according to the manufacturer’s instructions. The purity and concentration of the extracted RNA was determined spectrophotometrically using a Nanodrop 2000 (Thermo Fisher Scientific Inc., Waltham, MA, USA). Total RNA (500 ng) was reverse transcribed using the Transgen EasyScript^®^ one-step RT-PCR supermix (Beijing TransGen Biotech Co., Ltd., Beijing, China) according to the manufacturer’s instructions. Samples were subjected to an initial incubation at 45°C for 30 min for cDNA synthesis, followed by PCR amplification, using gene-specific primers (Table 2), 94°C for 10 min followed by 40 cycles of 94°C for 20 s, 20 s at the annealing temperature of primers, and 30 s at 72°C. All amplifications were done in a C1000 Touch™ Thermal Cycler (BioRad, Hercules, CA, USA). The amplicons were visualized on 1.5% agarose using a UVP BioDoc-It™ Imaging system (Upland, CA, USA). The intensity of the amplicon bands was analyzed using Image J software (25). The results were presented as the relative expression of the gene to β-actin, a reference gene.

**Table 2:** Sequences of gene-specific primers.

Gene	Sequence (5′–3′)	Template
β-Actin	Forward: AGCCATGTACGTAGCCATCC Reverse: CTCTCAGCTGTGGTGGTGAA	NM_031144.3
GK	Forward: CATATGTGCTCCGCAGGACTA Reverse: CTTGTACACGGAGCCATCCA	NM_001270850.1
HK	Forward: ACCCACGAAACAACACCATCA Reverse: GACGTACAACAATGGCTCACTAAAG	NM_012734.1
PFK-1 (Liver)	Forward: TTACCGATCACCCTCGTTCCT Reverse: TTCCCCTTAGTGCTGGGATCT	NM_013190.4
SOD	Forward: AGGATTAACTGAAGGCGAGCAT Reverse: TCTACAGTTAGCAGGCCAGCAG	NM_017050.1
Gpx	Forward: AAGGTGCTGCTCATTGAGAATG Reverse: CGTCTGGACCTACCAGGAACTT	NM_030826.4

### Statistical Analysis

The results were expressed as the mean ± SEM, and the differences between treated and control groups were statistically assessed using one-way ANOVA followed by *post hoc* Dunnett’s multiple range test with the aid of GraphPad Prism version 5.00 for windows (GraphPad Software, La Jolla, CA, USA). The results were considered significant whenever *p* < 0.05.

## Results

**Figure 1:**
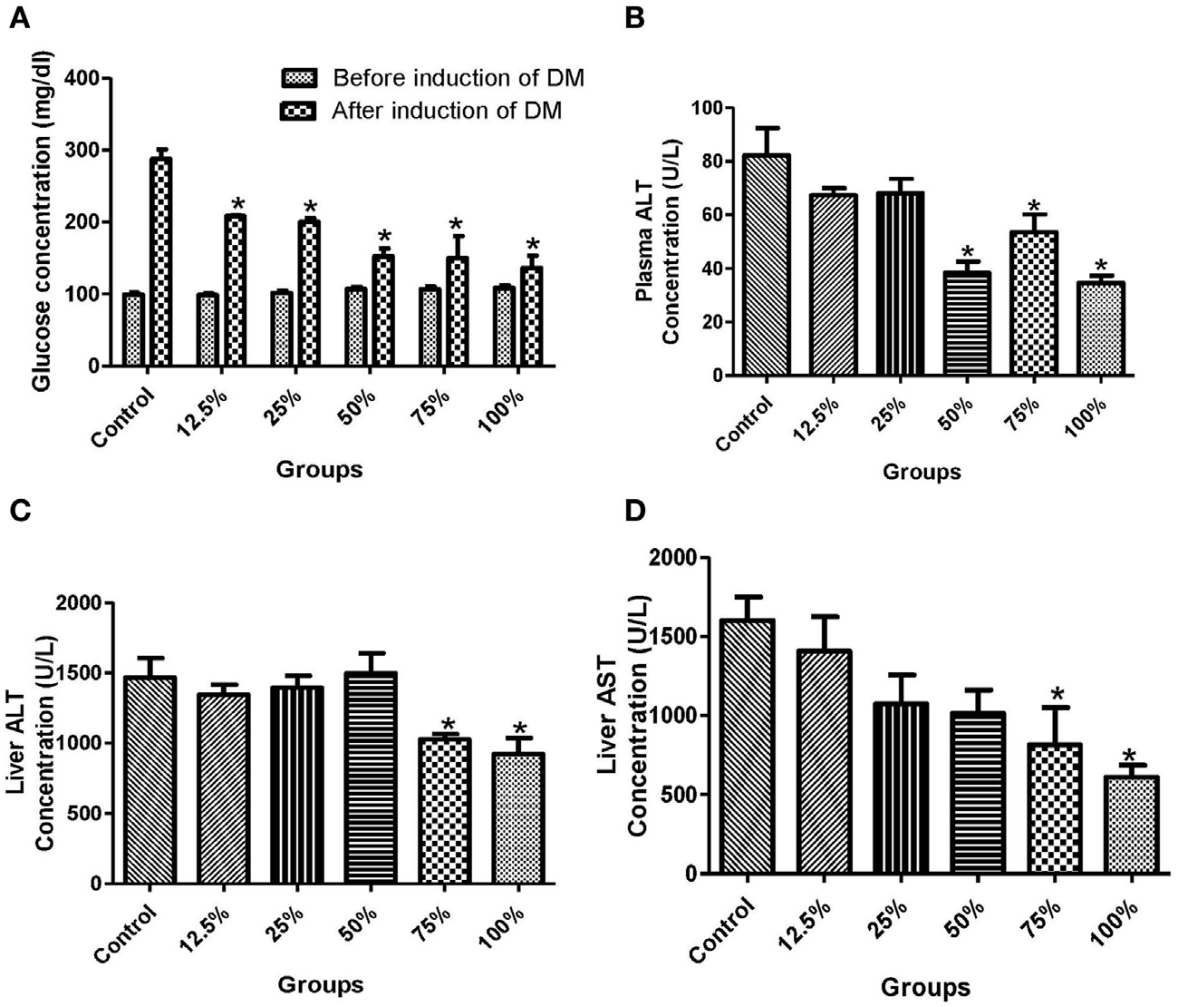
Effects of fermented sorghum diet on **(A)** the blood glucose levels before and after induction of diabetes, **(B)** the activities of alanine transaminase (ALT) in the erythrocyte, **(C)** the activities of ALT in the liver, **(D)** the activities of aspartate transaminase (AST) in the liver of alloxan-induced diabetic rats. All values are represented as the means ± SEM (*n* = 5). *Significant differences at *p* < 0.05 from the control group.

**Figure 2:**
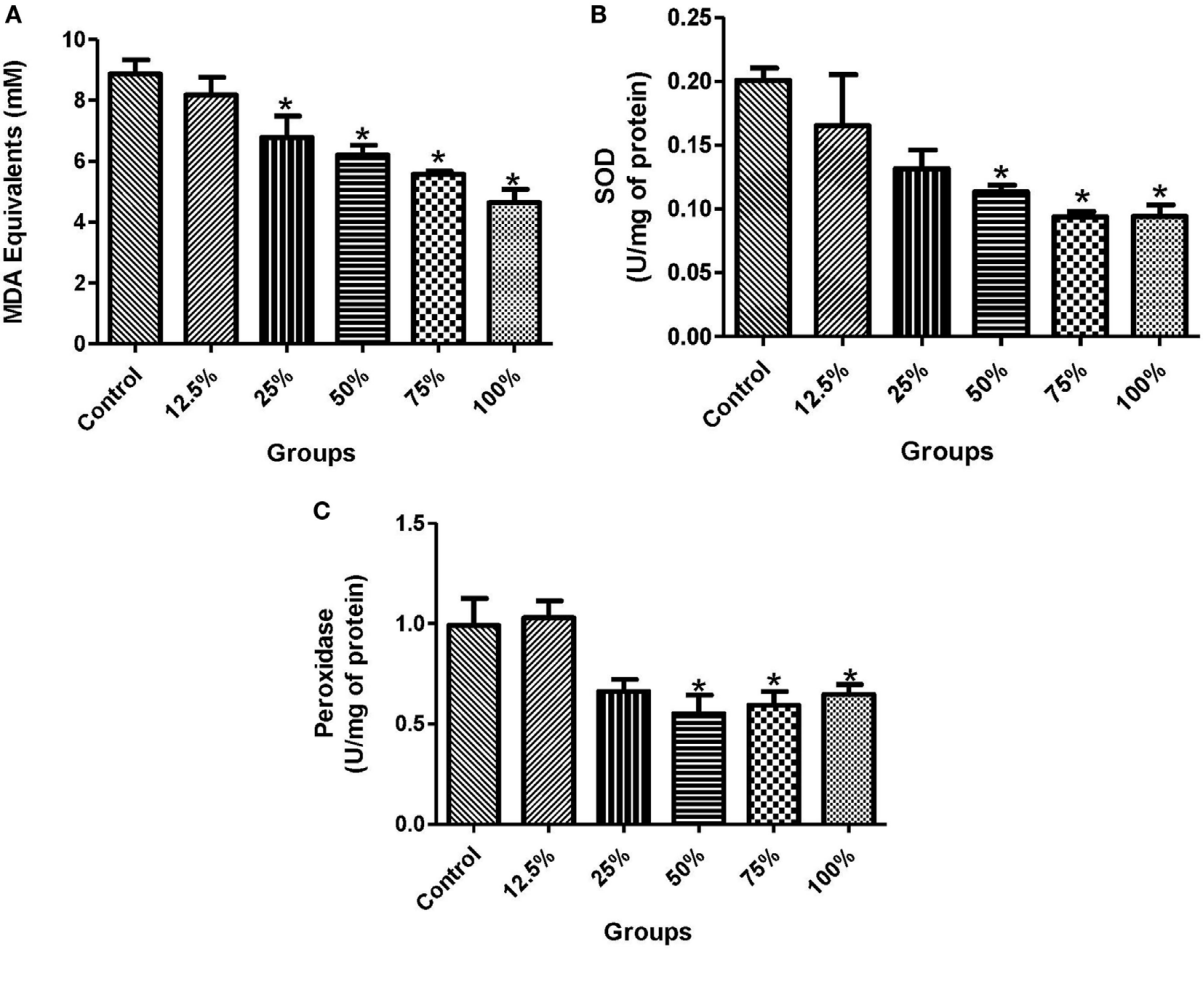
Effects of fermented sorghum diet on **(A)** MDA equivalents, **(B)** the activities of superoxide dismutase, and **(C)** the activities of peroxidase in the liver of alloxan-induced diabetic rats. All values are represented as the means ± SEM (*n* = 5). *Significant differences at *p* < 0.05 from the control group.

The administration of all the five sorghum diets significantly reduced (*p* < 0.05) the blood glucose levels compared with the control (Figure 1A). In the plasma, the activities of ALT were significantly reduced (*p* < 0.05) by the preconsumption of the three highest diets concentration (100, 75, and 50%) compared with the control (Figure 1B). The preconsumption of 100 and 75% sorghum diets significantly reduced (*p* < 0.05) the activities of ALT and AST in the liver compared with the control (Figures 1C,D). The preadministration of all the four highest concentration of the diets (100, 75, 50, and 25%) significantly decreased (*p* < 0.05) the level of lipid peroxidation compared to the control (Figure 2A). However, only the three highest sorghum diet concentrations (100, 75, and 50%) significantly reduced (*p* < 0.05) the activities of SOD and Px compared to the control (Figures 2B,C).

**Figure 3:**
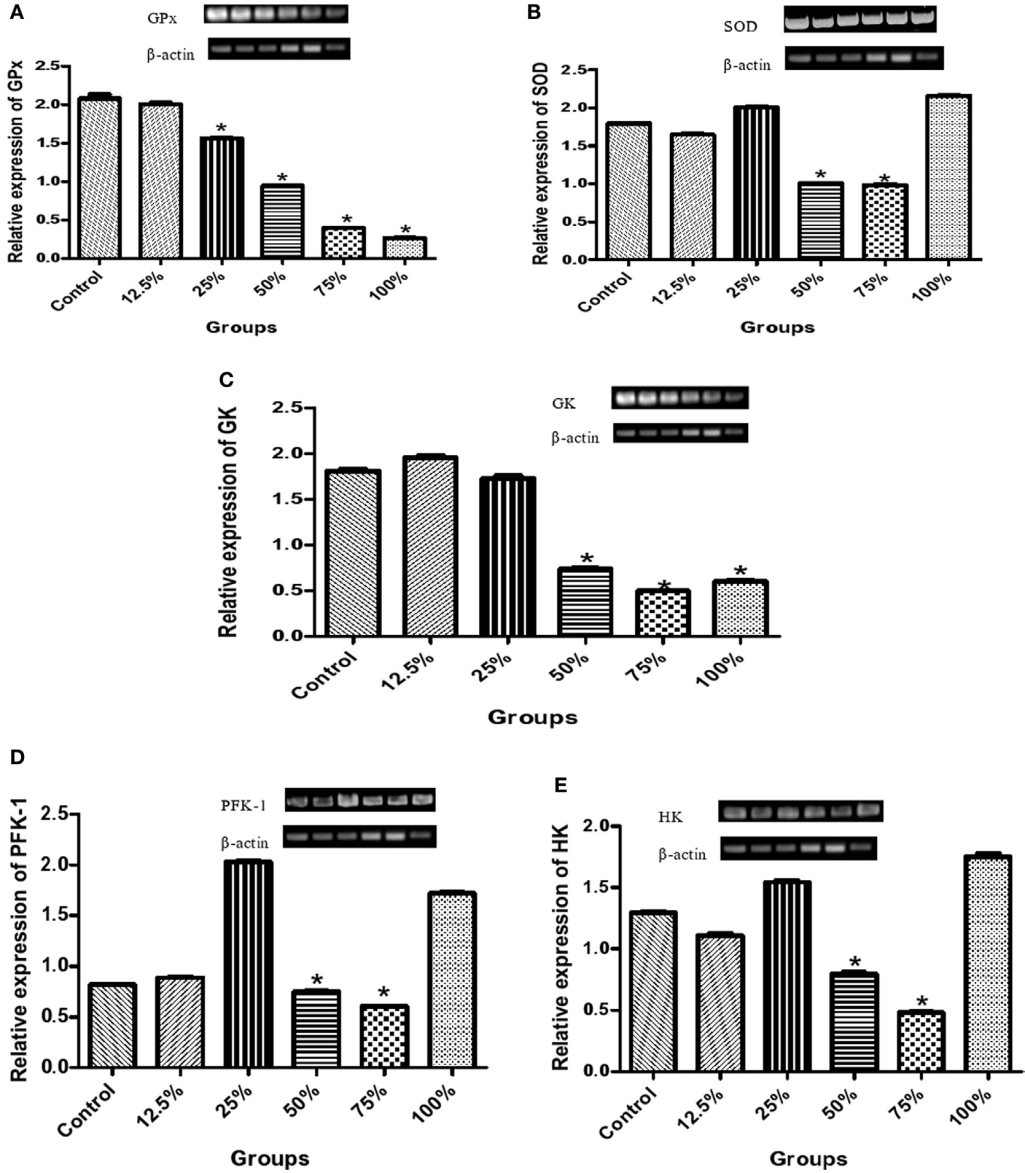
Effects of fermented sorghum diet on relative expression of hepatic **(A)** glutathione peroxidase, **(B)** superoxide dismutase, **(C)** glucokinase, **(D)** phosphofructokinase-1, and **(E)** hexokinase genes in alloxan-induced diabetic rats. All values are represented as the means ± SEM (*n* = 5). *Significant differences at *p* < 0.05 from the control group.

The preconsumption of the three highest sorghum diet concentrations (100, 75, and 50%) also significantly reduced (*p* < 0.05) the relative expression of glutathione peroxidase (GPx) compared to the control (Figure 3A), while it was only the two highest diet concentrations (75 and 50%) that significantly reduced (*p* < 0.05) the relative expression of SOD compared to the control group (Figure 3B). There was a significant decrease (*p* < 0.05) in the relative expression of GK due to the preconsumption of the highest sorghum diet concentration (100, 75, and 50%) compared with the control (Figure 3C). Also, a significant decrease (*p* < 0.05) in the relative expression of PFK-1 and hexokinase (HK) was observed by the preconsumption of mainly 75 and 50% of the diet concentration compared to the control (Figures 3D,E).

## Discussion

Our study showed that the consumption of the FSDs by the experimental rats for 8 weeks did not alter blood glucose levels (Figure 1A). Instead, it prevented drastic increase in the glucose levels following the administration of alloxan. This implies that the diets controlled homeostasis and stabilized energy utilization. Antinutritional factors in sorghum such as tannins, phytic acids, and phenolic acids usually slow down starch digestion by reducing the activity of digestive enzymes (11). More so, fermentation enhances the bulk density, digestibility, and phytochemical content of sorghum (26). The phytochemical contents of the extracts of sorghum had been implicated in the inhibition of α-amylase and α-glucosidase activities, which also improved the blood glucose levels (9).

This study also revealed that the regular consumption of the diets as depicted in the pretreatment of experimental rats before alloxan administration is efficient in reducing liver dysfunction in a dose-dependent manner (Figures 1B–D). Our finding was also similar to earlier reports that used the same/or slightly different experimental design (7, 9, 10, 27). Alloxan is known to increase glucose toxicity by destroying the pancreatic β-cells and reducing insulin release while causing hepatic malfunctioning concurrently (28). This may lead to liver injury and hence increased liver dysfunction indices such as ALT, AST, and ALP activities (28). The decrease in the activities of these transaminases in the liver and plasma due to treatment such as consumption of sorghum diet is a pointer to improved hepatic function (Figures 1B–D). The hepatoprotective activity of the diets as reflected in this study may be attributed to the antioxidant properties of its phytochemical constituents reported in sorghum cultivars (29). A recent study revealed that fermented sorghum extract is rich in bioactive compounds such as caffeic acid, hesperidin, quercetin, and rosmarinic acid (15). Our result is consistent with studies that have reported that fermented products of red ginseng and mung bean have antioxidant and hepatoprotective properties due to their phytochemical content (30, 31).

In evaluating the *in vivo* antioxidant potential of the sorghum diets, we discovered that the control group had a higher level of MDA concentration and increased activities of SOD and Px in the liver than the groups pretreated with the diets following the administration of alloxan to the experimental rats (Figures 2A–C). In diabetes, glucose toxicity usually increases the generation of ROS and thus, a remarkable increase in the activities of enzymatic and non-enzymatic antioxidant defense system (5). Interestingly, the reduced activity of antioxidant enzymes due to pretreatment with the diets in our study indicated that consumption of the diets protected the liver of rats from a chain of reaction caused by ROS, thereby reducing oxidative stress in a dose-dependent manner. The levels of ROS formed were normalized in the group fed with the diets unlike in the control that had overwhelming ROS concentrations and increased antioxidant enzymatic activities (Figures 2A–C). This is in accordance with the findings of Hassan and Emam (32), who recorded reduction in the levels of lipid peroxidation products in diabetic rats pretreated with camel milk and G*inkgo biloba* extract. In addition, the expression of GPx and SOD genes were also remarkably increased in the control in a bid to scavenge ROS and prevent diabetic complications (Figures 3A,B). Therefore, the lower level of activation of the antioxidant systems in this study is a result of the reduced level of ROS due to the consumption of the diets. This study also shows a positive correlation between the gene expression and activities of the antioxidant enzymes in alloxan-induced diabetic rats (Figures 3A,B). The antioxidant and protective activity of the diets may be attributed to the antiradical properties of sorghum and the free radical scavenging activities of its polyphenolics (15, 33).

The influence of pretreatment of rats with the sorghum diets on the mRNA transcription of GK, HK, and PFK-1 genes in the liver was also assessed in other to understand its effect on modulation of glucose metabolism particularly glycolysis. Our findings revealed that the preconsumption of the 75 and 50% diets by rats decreased the relative expression of these three genes (GK, HK, and PFK-1) in the liver compared to the control (Figures 3C–E). GK performs the function of phosphorylating glucose to glucose-6-phosphate in both glycolytic and glycogenesis pathways, and thus, its overexpression reduces blood glucose levels, favoring these pathways. A downregulation of GK in the sorghum diet pretreated rats indicates that blood glucose was normalized, and as such, the normal function of glycolysis was not affected. The result of the blood glucose in this study (Figure 1A) further substantiates this claim implying that elevated blood glucose in the control led to the rapid phosphorylation of glucose, favoring increased glycolysis in the liver. PFK-1 is the rate limiting enzyme of glycolysis, and our findings indicate that this enzyme was suppressed by the consumption of the two lowest concentrations of the diets (12.5 and 25%) in a similar pattern observed in the control. Overall, the diets did not promote glucose production or utilization in the liver *via* the upregulation of the expressions of GK, HK, and PFK-1, instead the diets protected the liver by normalizing blood glucose levels. It was observed that 100% diet raised the gene expression of PFK-1, SOD, and HK and we may attribute it to the saturation of the active site of the enzyme by the excess supply of the diet, thus the changes seen in the kinetic behavior of these enzymes. Earlier reports on the posttreatment of extract of *Vernonia amygdalina* and *Ficus deltoidea* have indicated changes in the expression of key glucose modulatory enzymes in diabetic rats (24, 34), whereas this study was focused on the effect of pretreatment of sorghum diets on diabetic rats.

## Conclusion

This study demonstrated that fermented sorghum product may be useful in preventing DM. At the molecular level, the regular consumption of FSD had blood glucose-lowering properties, attenuated the degree of oxidative stress, and consequently suppressed the expression of key glycolytic target genes in the liver of alloxan-induced diabetic rats. We recommend that more biomarkers of oxidative stress assessing protein, DNA/RNA damage, and insulin levels can be evaluated in further studies as these would add strength to our observations.

## Ethics Statement

This study was carried out in accordance with the recommendations of ethical committee of Covenant University, Ota, Nigeria, with the reference number CU/BIOSCRECU/BIO/2015/010. The protocol was approved by the ethical committee of Covenant University.

## Author Contributions

TO, IA, and OO designed the research. TO and MO conducted the research. SR assisted in the feed composition and gene expression study. TO, IA, and OO analyzed the data. TO wrote the paper. All authors read and approved the final manuscript.

## Conflict of Interest Statement

The authors declare that the research was conducted in the absence of any commercial or financial relationships that could be construed as a potential conflict of interest.
